# Targeting PEPT1: a novel strategy to improve the antitumor efficacy of doxorubicin in human hepatocellular carcinoma therapy

**DOI:** 10.18632/oncotarget.17117

**Published:** 2017-04-15

**Authors:** Yanxia Gong, Xiang Wu, Tao Wang, Jia Zhao, Xi Liu, Zhi Yao, Qingyu Zhang, Xu Jian

**Affiliations:** ^1^ Department of Gastroenterology, Tianjin Medical University General Hospital, Tianjin, 300052, China; ^2^ Department of Gastroenterology, Tianjin Nankai Hospital, Tianjin, 300100, China; ^3^ Central Laboratory, Tianjin Medical University General Hospital, Tianjin, 300052, China; ^4^ Department of Immunology, School of Basic Medical Science, Tianjin Medical University, Tianjin, 300070, China

**Keywords:** proton coupled oligopeptide transporter 1, doxorubicin, hepatocellular carcinoma (HCC), target therapy, toxicity

## Abstract

Proton coupled oligopeptide transporter 1 (PEPT1) is a member of the peptide transporter superfamily and plays important role in the absorption of oligopeptide and peptidomimetic drugs. Our previous research verified that PEPT1 expressed specifically in human Hepatocellular carcinoma (HCC) tissue and cell lines and showed potential transport activity to be a new candidate of the tumor therapeutic target. In this study, we aim to explore the feasibility of a novel tumor target therapeutic strategy: Targeting PEPT1 to improve the antitumor efficacy of Doxorubicin in human HCC therapy. First, Doxorubicin was conjugated with Glycylglycylglycine (Gly-Gly-Gly) − a tripeptide which was known as the substrate of PEPT1 and characterized by HPLC and MS successfully. Doxorubicin-tripeptide conjugate was then observed to clarify the target delivery by PEPT1 and the antitumor effect on human hepatocarcinoma *in vivo* and *in vitro*. Furthermore, the improvement of the toxic and side effect of Doxorubicin after conjugation was also evaluated by some biochemical tests. Our results reveal that targeting PEPT1 may contribute to the efficient delivery of Doxorubicin to hepatocarcinoma cells and the reduction of drug toxicity. PEPT1 has the prospect to be a novel target of HCC therapy.

## INTRODUCTION

HCC is one of the most common malignant tumors worldwide. China has the high morbidity and mortality of HCC and the number of the HCC patients in China account for 54% of the world [1]. Due to the occult onset and difficult to early diagnosis, most of the HCC patients missed the optimal operating timing. Thus, non-operative therapy including chemotherapy is an important treatment strategy for advanced HCC. But the chemotherapeutic agents are relatively ineffective and result in many serious toxic and side effects. Doxorubicin is a traditional chemotherapeutic agent for a wide variety of cancers. Despite of being highly effective, the use of Doxorubicin has many limitations due to the significant toxicity and side effects occurred during and after HCC treatment. These toxicities usually affect heart, brain, bone marrow and the consequences of these toxicities are often very apparent and will lasted for many years after treatment [2]. Therefore it is urgent to explore the new therapeutic strategy to improve the efficacy of the antitumor agents.

In recent researches, Target therapy has become a potent therapeutic strategy for tumor which delivered the antitumor drug to the lesion directly and accurately and has the advantage of less toxicity to the normal tissues. Therefore some antitumor targets such as nanoparticle, tumor specific antigen, tumor specific receptor are under investigating and play key role in tumor target therapy strategy [3, 4]. PEPT1 is one of the four members of peptide transporter super family in mammalian cells, which is expressed predominantly in intestine and is now recognized as the major route by which dietary nutrients (di/tripeptide) or peptidomimetic drugs were absorbed [5]. Because of the capacity of PEPT1 to transport a broad spectrum of substrates, it has made an attractive target for drug delivery. Despite of intestine expression, PEPT1 also be found in some carcinomas such as pancreatic carcinoma, prostate cancer, gastric cancer et al [6, 7]. Our recent study also verified that PEPT1 can be specific expressed in human hepatocarcinoma tissue and cell lines and has the transport activity to mediate the delivery of oligopeptide or peptidomimetic molecules. Therefore we speculate that PEPT1 has the prospect to be a promising target for tumor target therapy. Those reports involving in PEPT1 mostly concentrate on the molecular expression and functional activity investigation but few focus on the application of PEPT1 in tumor target therapy. The feasibility and effect of utilizing PEPT1to specific deliver the antitumor chemotherapy agents to the target cancer cells and reducing the side effect on the normal site was fewer reported.

In this study, we aim to explore the feasibility of a novel tumor target therapeutic strategy: Targeting PEPT1 to improve the antitumor efficacy of Doxorubicin in human HCC therapy. We conjugate Doxorubicin with a tripeptide ligand − Gly-Gly-Gly which is a known substrate of PEPT1 to make it be recognized by PEPT1 and transported into the HCC cells. Then we investigated the antitumor effect of Doxorubicin-tripeptide conjugate on HCC *in vitro* and *in vivo* and observed the improvement of the side effects. According to our study we speculate that targeting PEPT1 may be a new and efficient strategy to improve the antitumor effect and overcome the drug side effect of Doxorubicin on HCC therapy. PEPT1 has the prospect to be a novel target of HCC therapy.

## RESULTS

### The molecular expression and transport activity of PEPT1 in HCC

**Figure 1:**
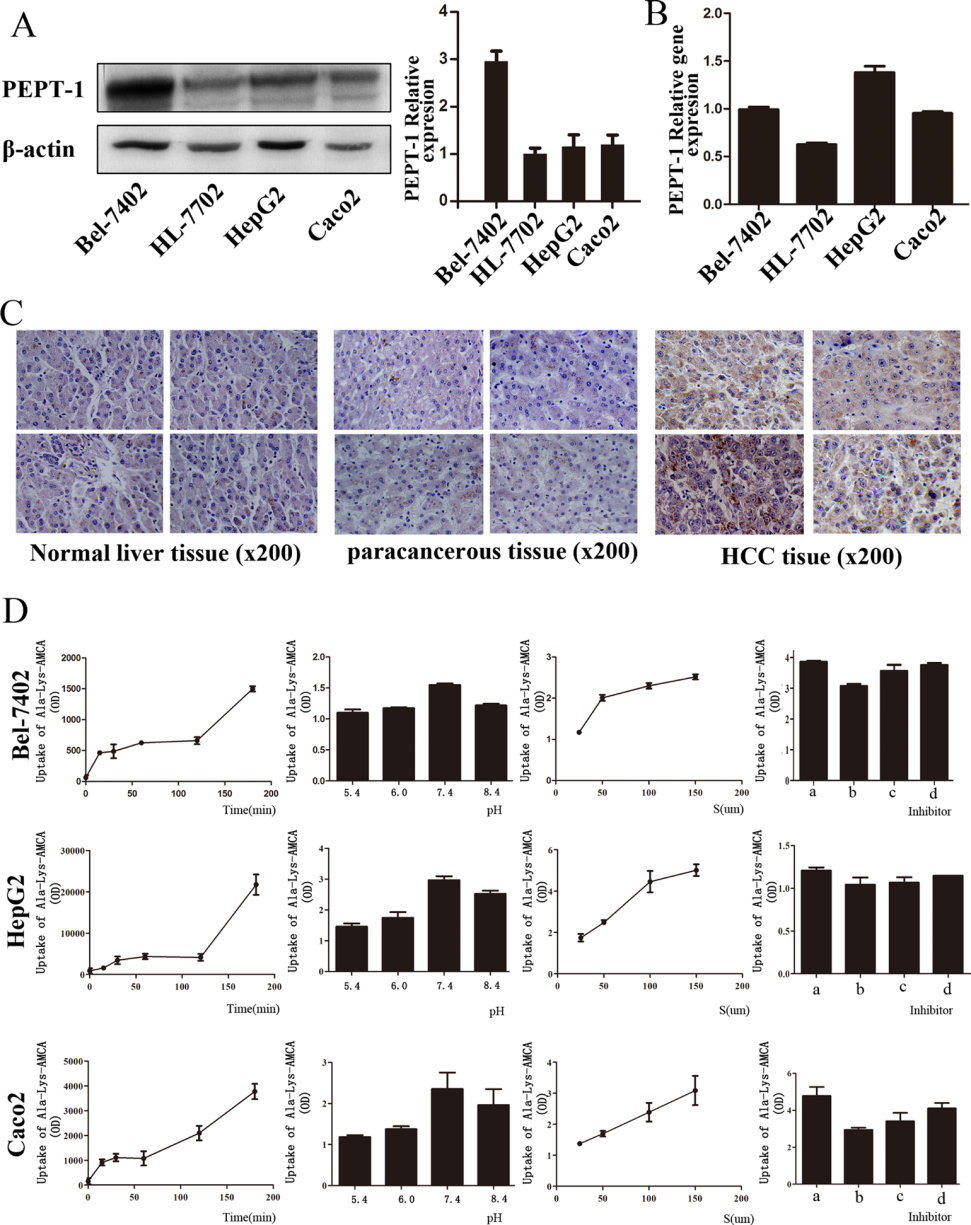
The previous research results were presented The expression of PEPT1 in liver normal cell line HL-7702, hepatocarcinoma cell lines (Bel-7402, HepG2) and human colon cancer cell line Caco-2 by Western blotting (**A**) and Real-time RT-PCR (**B**). The expression of PEPT1 based on immunohistochemistry in different liver tissues (**C**, **D**): The uptake of Ala-Lys-AMCA under different conditions, from left to right was the uptake at different times (0, 15, 30, 60, 120, 180 min), at different pH values (5.4, 6, 7.4, 8.4), at different concentrations (25 μmol/L, 50 μmol/L, and 150 μmol/L) and at diffrernt inhibitors (a: without inhibitor, b with inhibitor Gly-Sar, c: with inhibitor Gly-Sar, Gly-Gln, d: with inhibitor Gly-Gly-Gly).

Our study reveals that the protein and mRNA expression of PEPT1 were higher in HCC cells Bel-7402 and HepG2, as well as in Caco-2 cells, compared with normal liver cell line HL-7702, according to Western blotting and real-time RT-PCR (Figure 1A and 1B). The protein expression of PEPT1 in HCC tissues was higher than that observed in adjacent tissues and normal liver tissues (*P* = 0.0193 and *P* = 0.0057, respectively), which was detected in our former work with a total of 82 cases of human HCC tissue (Figure 1C). Additionally, PEPT1 expressed in HCC cells shows the oligopeptide transport activity. The uptake of Ala-Lys-AMCA, a classic substrate of PEPT1 was time-dependent and concentration-dependent and shows competitive inhibition by three known substrates of PEPT1 in Bel-7402, HepG2 and Caco-2 cells (Figure 1D).

### Synthesis and identification of Doxorubicin-tripeptide conjugate

**Figure 2:**
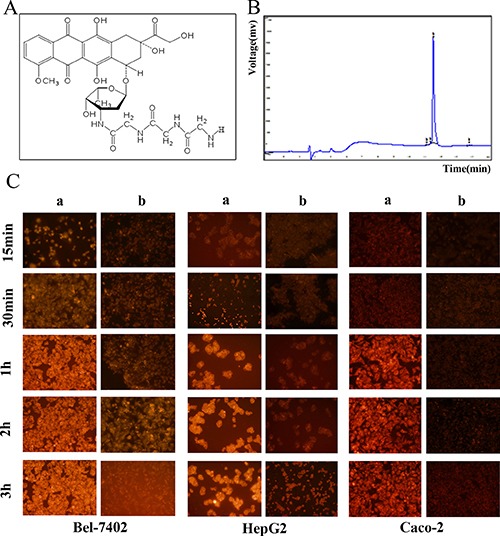
(**A**) The structure of Doxorubicin-tripeptide conjugate. (**B**) The identification of Doxorubicin-tripeptide conjugate by HPLC. (**C**) The drug uptake of HCC cells by fluorescence microscope, a: Doxorubicin-tripeptide conjugate, b: Doxorubicin.

Doxorubicin was successfully conjugated with a tripeptide Gly-Gly-Gly, which is a substrate of PEPT1. Doxorubicin-tripeptide conjugate was purified and identified by HPLC and MS. The purity of Doxorubicin-tripeptide conjugate is 98.00%. The structure and HPLC chart were as shown below (Figure 2A and 2B).

### Inhibitory effect of doxorubicin-tripeptide conjugate on HCC cells by MTT

The IC50 of Doxorubicin-tripeptide conjugate was 0.05 mg/ml detected by MTT. The inhibition effect of Doxorubicin-tripeptide conjugate have showed dose independent. According to the dose equivalent principle of Doxorubicin and the results of MTT, the drug concentration chosen *in vitro* cell experiment was Doxorubicin-tripeptide conjugate 0.05 mg/ml and Doxorubicin 0.01 mg/ml (Table 1).

**Table 1:** The Inhibitory effect of drugs on tumor cells by MTT (mg/ml)

	Bel-7402	HepG2	Caco2
**doxorubicin**			
0.005	0.3878	0.3456	0.3461
0.01	0.4913	0.4217	0.5002
0.02	0.6121	0.5732	0.5793
0.04	0.7001	0.6657	0.6234
0.08	0.7982	0.8791	0.7894
IC50	0.0138	0.0147	0.0154
**doxorubicin-tripeptide conjugate**			
0.01	0.1402	0.1543	0.1673
0.02	0.2602	0.2931	0.3657
0.04	0.4837	0.4765	0.4231
0.08	0.5834	0.5643	0.5432
0.16	0.6843	0.6873	0.6453
IC50	0.0525	0.0514	0.0529

### The observation of doxorubicin-tripeptide conjugate uptake by HCC cells under fluorescence microscope

Doxorubicin-tripeptide conjugate and Doxorubicin can emit red fluorescence. Based on the above findings, we confirmed that these two drugs can be transported into HCC cells as well as Caco-2 cells at the periphery of the nucleus with red fluorescence aggregation, and the longer the action time, the stronger the fluorescence density and strength. Compared with Doxorubicin, in the same drug action time, the Doxorubicin-tripeptide conjugate had a more significant effect (Figure 2C).

### The concentration analysis of Doxorubicin in HCC by HPLC

**Figure 3:**
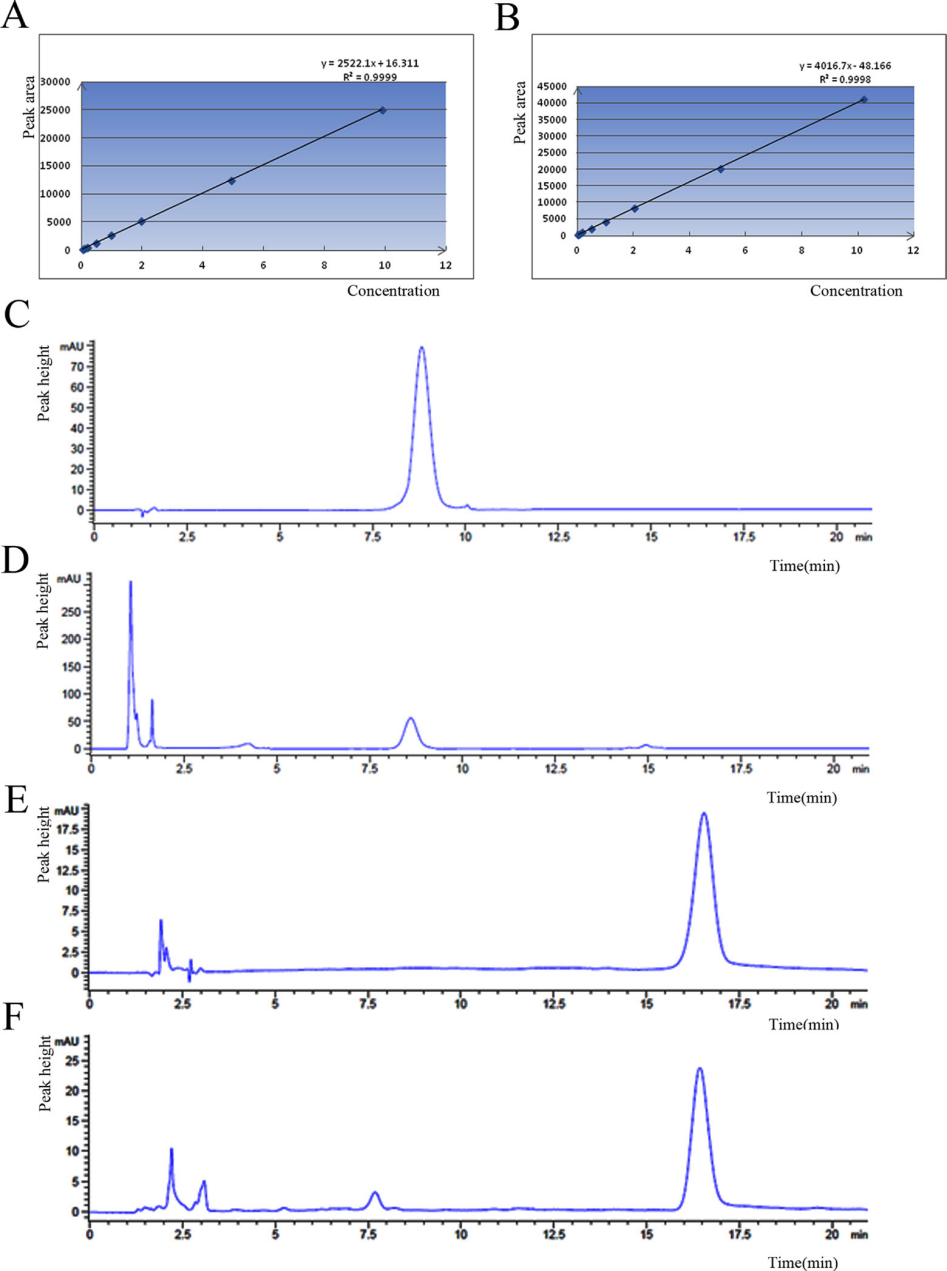
The standard curve of drugs, (**A**) Doxorubicin-tripeptide conjugate, (**B**) Doxorubicin. The Analysis by HPLC, (**C**) the standard product of Doxorubicin-tripeptide conjugate, (**D**) The intracellular extraction of Doxorubicin-tripeptide conjugate, (**E**) the standard product of Doxorubicin, (**F**) The intracellular extraction of Doxorubicin.

The chromatographic peak was located in 16.5 min of Doxorubicin and 8.7 min of Doxorubicin-tripeptide conjugate. The regression equation of Doxorubicin-tripeptide conjugate was as bellow: y = 4016.x–48.16 (linearity range 0.051~10.2 ug.ml^–1^, *R*^2^ = 0.999), while the regression equation of Doxorubicin revealed as follows: y = 2522.x + 16.31(linearity range 0.0495~9.9 ug.ml^-1^, *R*^2^ = 0.999). The following table showed the concentration ns of these two drugs in Bel-7402, HepG2 and Caco2 cells measured by HPLC, which presented that Doxorubicin-tripeptide conjugate had a higher drug concentration (Figure 3, Table 2).

**Table 2:** The detection of drug concentration in tumor cells (μg/ml)

	Doxorubicin-tripeptide conjugate group	Doxorubicin
**Bel-7402**	3.08 ± 0.03	2.42 ± 0.03
**HepG2**	2.94 ± 0.02	2.32 ± 0.03
**Caco2**	3.09 ± 0.03	2.73 ± 0.04

SPSS version 16.0 was used for all calculations. The differences between Adriamycin peptide conjugate and Adriamycin were all significant. *P* was 2-sided and *P* < 0.05.

### Uptake, dynamic and competitive inhibition test

**Figure 4:**
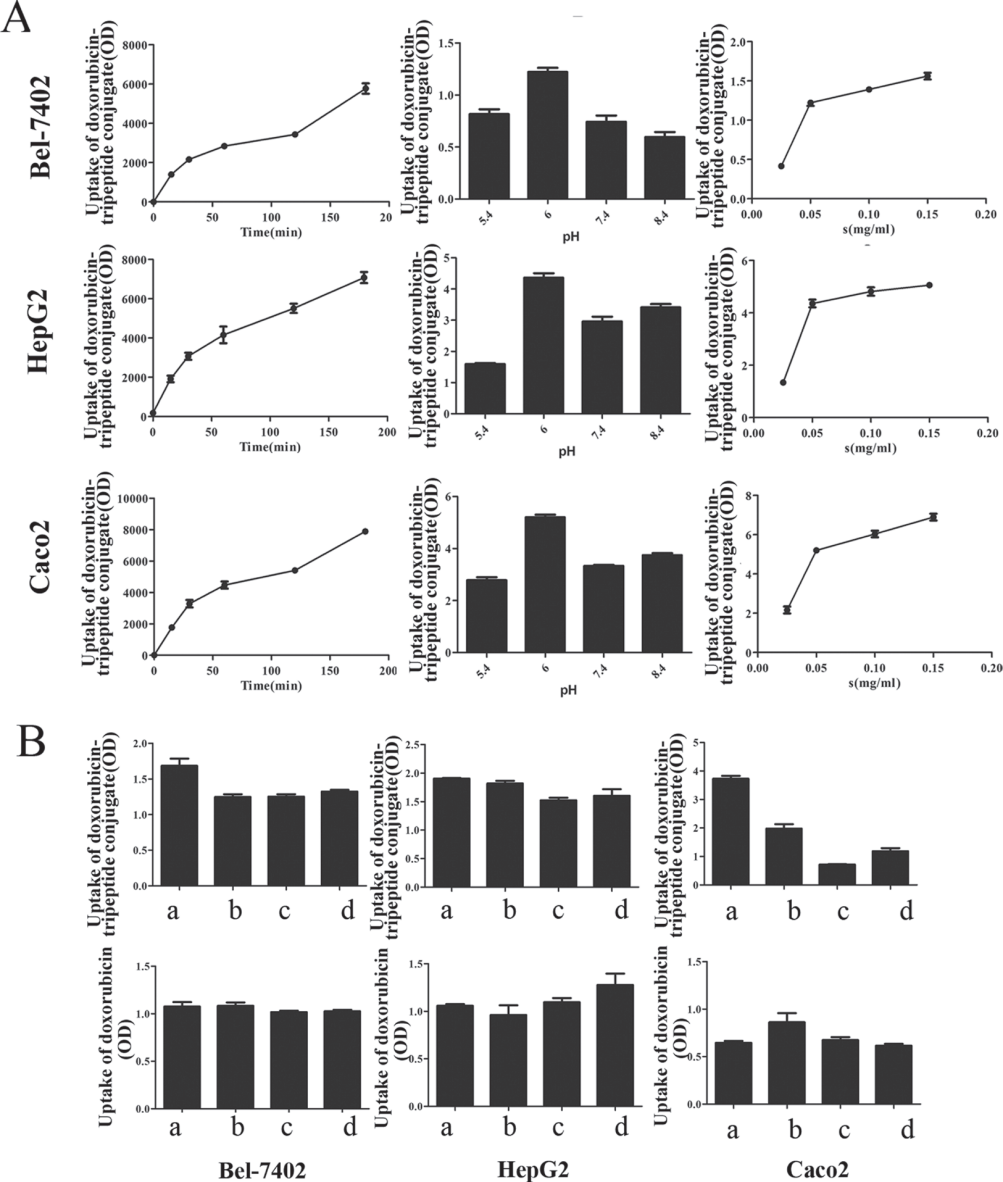
(**A**) The uptake of Doxorubicin-tripeptide conjugate under different conditions, from left to right was the uptake at different times (0, 15, 30, 60, 120, 180 min), at different pH values (5.4, 6, 7.4, 8.4), at different concentrations (25 μmol/L, 50 μmol/L, and 150 μmol/L). (**B**) The competitive inhibition test of drugs. The above graph: Doxorubicin-tripeptide conjugate, The below graph: Doxorubicin, a: without inhibitor, b with inhibitor Gly-Sar, c: with inhibitor Gly-Sar Gly-Gln, d: with inhibitor Gly-Gly-Gly.

The uptake of Doxorubicin-tripeptide conjugate was time-dependent and also concentration-dependent, but not pH-dependent. The maximum uptake occurred at a pH value of 6.0 (Figure 4A). Moreover, the uptake was significantly decreased by the presence of inhibitors of Gly-Sar, Gly-Gln or Gly-Gly-Gly in Doxorubicin-tripeptide conjugate, but not in Doxorubicin (Figure 4B).

### Animal study

### Antitumor effects of Doxorubicin-tripeptide conjugate on mice in survival quality, survival time and body weight

### Survival quality

The following observations were made regarding the general condition of group A mice: Mice showed slow activity, flagging spirit, loose and dull skin, decreased appetite, varying degrees of weight loss with the development of observation period. Group B appeared lethargy, vomiting and loss of appetite administered within 3 days, then gradually restored, but with continued treatment resulted in low spirits, little food intake and cachexia significantly. The survival quality of group C of mice was the best. Some mice appear to have an increased body weight and food intake. The above was more significant in high dose administration.

### Survival time

**Figure 5:**
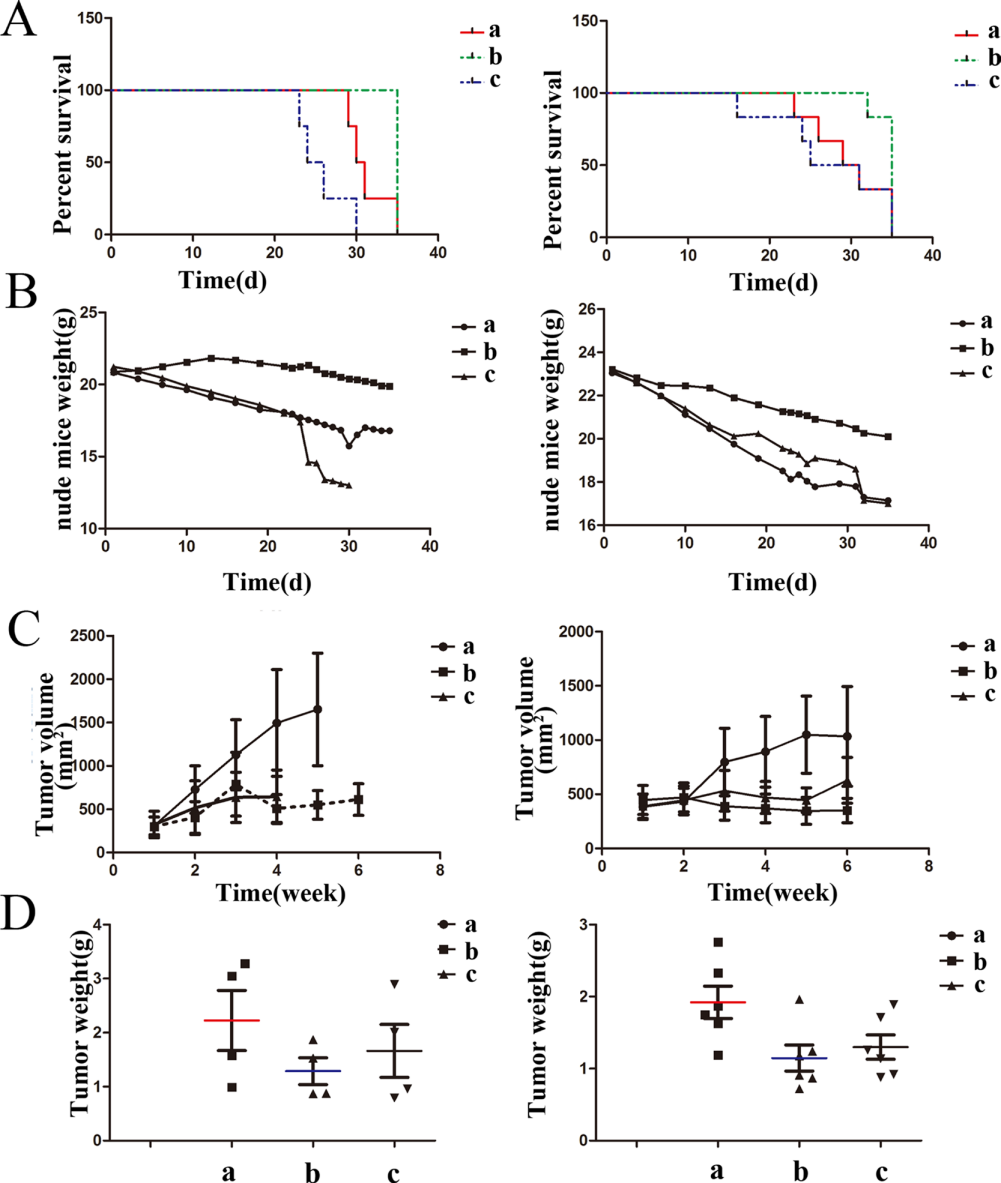
The effects of drugs on mice in survival time, mice weight and tumor inhibition (**A**) Mice survival was estimated by Kaplan-Meier survival analysis, The Changes in mice weight (**B**) and tumor volume (**C**) during the treatment period, (**D**) The tumor weight at the end of treatment. a: Control group, b: Doxorubicin-tripeptide conjugate group, c: Doxorubicin group.

In the low dose group (HepG2), the mice in group B had no death during the treatment of 5W, while group C with 3 deaths and 1 death respectively in the treatment of 3W+ and 4W+, group A with 3 deaths in the treatment of 4W+. The survival time was 29~35 d (median 32d) in group A, 23~30 d (median 26.5 d) in group C and 35 d (median 35 d) in group B. The survival time of group B was 8.57% (3.0 d) longer than that of the group A and 24.28% (8.5 d) longer than that of the group C. In the high dose group (Bel-7402), The mice in group B had 1 death during the treatment of 4W+, while group C with 1 death and 2 deaths and 1 death in the treatment of 2W+, 3W+ and 4W+ respectively, group A with equal 2 deaths in the treatment of 3W+ and 4W+. The survival time was 23~35 d (median 29 d) in group A, 16~35 d (median 25.5 d) in group C and 32~35 d (median 33.5 d) in group B. The survival time of group B was 12.86% (4.5 d) longer than that of group A and 22.86% (8.0 d) longer than that of group C. The difference in survival time had statistical significance (*P* < 0.05) (Figure 5A).

### Mice weight

There was no difference in mice weight among three groups before treatment. After administrated, all group appeared different degrees of weight loss, among which group C were the most significant. There was a weight increase in group B at the early stage of treatment, but a weight decreased significantly at the late stage of treatment. Additionally, the dosage increased the above changes were more obvious (Figure 5B, Table 3).

**Table 3:** The inhibitory effects of drugs on mice with HCC

	Mice weight (g)		Tumor volume (cm^2^)		Tumor volume (cm^2^)	Tumor weight (g)	Inhibition rate (%)
	Before treatment	After treatment	Before treatment	After treatment			
**The HepG2 HCC mice**							
**A**	20.83 ± 4.27	16.53 ± 2.40	0.31 ± 0.20	1.65 ± 1.29	1.09 ± 0.82	2.07 ± 0.95	
**B**	20.88 ± 5.37	19.90 ± 3.71	0.30 ± 0.22	0.61 ± 0.36	0.46 ± 0.23	1.46 ± 0.61	42.13^b^
**C**	21.23 ± 4.97	17.58 ± 4.21	0.32 ± 0.30	0.64 ± 0.61	0.50 ± 0.46	1.66 ± 0.98	25.28
**The Bel-7402 HCC mice**							
**A**	23.05 ± 1.62	17.45 ± 0.83^d^	0.38 ± 0.29	1.10 ± 0.89	0.49 ± 0.37	1.92 ± 0.55	
**B**	23.22 ± 1.57	20.13 ± 1.09^a^	0.44 ± 0.33	0.35 ± 0.27	0.25 ± 0.17	1.15 ± 0.44^a^	40.33^b^
**C**	23.15 ± 1.60	18.07 ± 2.01^d^	0.38 ± 0.28	0.42 ± 0.33	0.27 ± 0.16	1.30 ± 0.41	32.35^c^

SPSS version 16.0 was used for all calculations. ^a^Significant difference compared to group A. ^b^Significant difference compared to group C. ^c^Significant difference between the HepG2 HCC mice vs. the Bel-7402 HCC mice. ^d^Significant difference between before treatment vs. after treatment. *P* was 2-sided and *P* < 0.05.

### Drug inhibition rate

The Doxorubicin-tripeptide conjugate can inhibit the growth of tumor. The tumor inhibition rates were 40.33%, 42.13% respectively comparing with the tumor inhibition rates of Doxorubicin 32.35% and 25.28% in the high dose group and low dose group. The subcutaneous tumors were dissected and weighed at the end of therapy, and the weight from high to low was the Control group (group A), the Doxorubicin-tripeptide conjugate group (Group C) and Doxorubicin group (group B). As the Doxorubicin-tripeptide conjugate dose increasing, the inhibition rate of it on HCC will increase (Figure 5C and 5D, Table 3).

### Drug distribution in different tissues of mice

**Figure 6:**
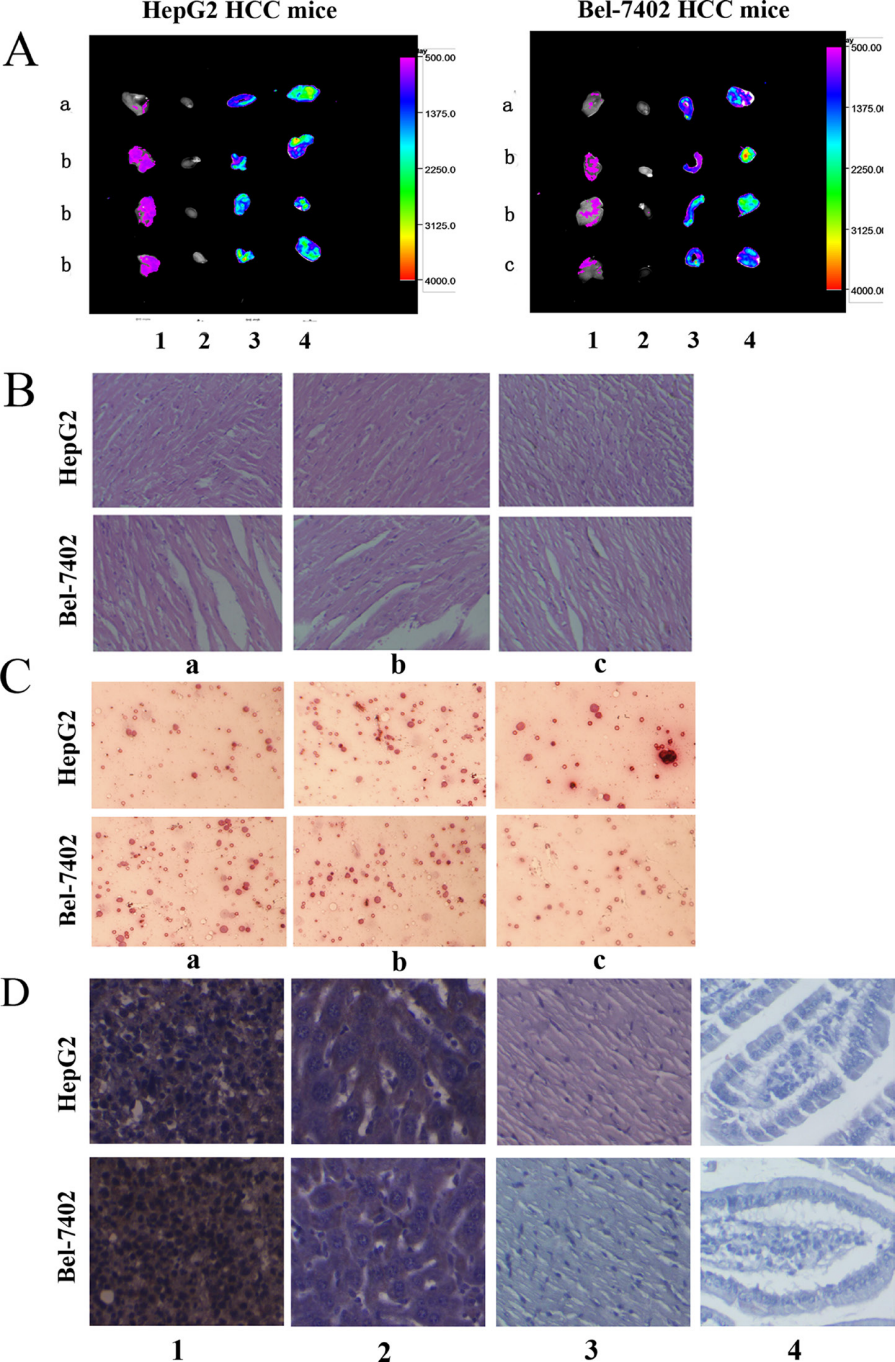
(**A**) The drug distribution in different tissues of mice of different group, 1: tumor, 2: liver, 3: heart, 4: jejunum. a: Control group, b: Doxorubicin-tripeptide conjugate group, c: Doxorubicin group. (**B**) The cardiac pathology in each group (×200), a: Control group, b: Doxorubicin-tripeptide conjugate group, c: Doxorubicin group. (**C**) The bone marrow smear in each group (×200), a: Control group, b: Doxorubicin-tripeptide conjugate group, c: Doxorubicin group. (**D**) The expression of PEPT1 in different tissues of mice (×200), 1: tumor, 2: liver, 3: heart, 4: jejunum.

In the Bel-7402 HCC mice (high dose group), it can be observed that Doxorubicin-tripeptide conjugate was mainly distributed in subcutaneous tumor tissue and liver metastatic tumor tissue and less in heart tissue compared with Doxorubicin group. In the HepG2 HCC mice (low dose group), due to the mice of Doxorubicin group were all death at the end of the treatment, only the control group and Doxorubicin-tripeptide conjugate group can be proceeded by Animal Imaging, the same can be seen that Doxorubicin-tripeptide conjugate was mainly distributed in subcutaneous tumor tissue and less in heart tissue compared with control group (Figure 6A).

### Cardiac and bone marrow index detection

Doxorubicin-tripeptide conjugate was less damage to the heart and bone marrow when compared with the Doxorubicin group by the detection of cardiac biochemical indexes, myocardial histopathology, peripheral blood routine and bone marrow smears (*P* < 0.05). When the drug dosage was increased, the Doxorubicin-tripeptide conjugate had more effective in cancer treatment with fewer side effects, while the tumor inhibition rate of the Doxorubicin was improved but associated with drug toxicity increased (Figure 6B and 6C, Table 4, Table 5).

**Table 4:** The detection of Cardiac indexes in each group

	AST (U/L)	CK (U/L)	CK-Mb (U/L)	LDH (U/L)	MDA (nmol/mg)	GSH-PX (U)	SOD (U/mg)
**The HepG2 HCC mice**							
**A**	734.25 ± 424.51	1164.50 ± 204.43	514.25 ± 99.13	5455.50 ± 1152.51	2.55 ± 0.44	745.72 ± 73.89	187.21 ± 13.72
**B**	637.75 ± 319.83	1070.75 ± 193.32	570.00 ± 99.07^b^	6116.75 ± 1136.22	3.16 ± 0.26	682.33 ± 51.09	164.15 ± 25.20
**C**	989.25 ± 145.74	1160.50 ± 164.82	797.00 ± 155.90^a^	6958.50 ± 991.50	3.97 ± 0.50	515.57 ± 146.57^a^	135.63 ± 16.22^a^
**The Bel-7402 HCC mice**							
**A**	567.00 ± 215.79	1021.83 ± 125.57	523.00 ± 222.09	4808.83 ± 1160.77	2.38 ± 0.32	754.34 ± 128.63	192.92 ± 10.69
**B**	720.83 ± 303.52^b^	1025.17 ± 182.34^b^	424.33 ± 189.91^b^	6517.00 ± 1956.59	2.78 ± 0.22^b^	700.50 ± 79.58^b^	171.71 ± 15.51
**C**	1400.83 ± 506.35^a^	1357.83 ± 504.33^ac^	823.17 ± 342.53	5811.67 ± 3290.44	3.43 ± 0.35^a^	462.36 ± 100.90^a^	151.60 ± 18.08^a^

SPSS version 16.0 was used for all calculations. ^a^Significant difference compared to group A. ^b^Significant difference compared to group C. ^c^Significant difference between the HepG2 HCC mice vs. the Bel-7402 HCC mice. *P* was 2-sided and *P* < 0.05.

**Table 5:** The detection of blood routine in each group

	WBC (×10^9^/l)	RBC (×10^12^/l)	PLT (×10^9^/l)
**The HepG2 HCC mice**			
A	12.78 ± 8.11	7.70 ± 1.47	192.50 ± 93.28
B	8.08 ± 3.40	8.03 ± 0.91	135.00 ± 69.55
C	4.90 ± 3.60	7.55 ± 1.33	72.53 ± 25.46 ^a^
**The Bel-7402 HCC mice**			
A	6.87 ± 2.20	7.53 ± 1.13	201.83 ± 82.97
B	7.05 ± 2.70^b^	7.50 ± 1.77	159.83 ± 74.74^b^
C	3.66 ± 2.58^a^	6.90 ± 2.32	81.33 ± 27.50^a^

SPSS version 16.0 was used for all calculations. ^a^Significant difference compared to group A. ^b^Significant difference compared to group C. ^c^Significant difference between the HepG2 HCC mice vs. the Bel-7402 HCC mice. *P* was 2-sided and *P* < 0.05.

### Expression of PEPT1 in different tissues of mice

Tumor, liver, heart and jejunum tissues of mice were taken for immunohistochemistry, and the expression of PEPT1 was further verified. It was found that there were more positive particles in the tumor tissues and partly liver tissues while less expression of the positive particles in the heart and jejunum tissues (Figure 6D).

## DISCUSSION

Primary hepatocellular carcinoma (HCC), which arises from liver cells or intrahepatic bile duct epithelial cells, is one of the most fatal malignant tumors worldwide, and its morbidity and mortality are increasing in recent years. Due to the high incidence of hepatitis B, China is also has the high incidence of HCC [11]. The preferred treatment for HCC is liver resection, but this treatment is restricted to the very early stages of HCC [12]. Due to the difficulty associated with early diagnosis, the rapid progression of the disease and the fact that the majority of patients have liver cirrhosis, few patients are able to undergo the operation, resulting in the majority of patients having a poor prognosis [13]. Thus, non-operative therapy is an important treatment strategy for advanced HCC [14, 15]. However, chemotherapy drugs are not specific to liver cancer cells, they may also affect normal cells and may result in serious adverse effects. Therefore, the currently available therapies offer limited benefits to patients. As a result, there is an urgent need for the development of novel, better therapeutic strategies. A targeted drug delivery system that can be used as an effective specific treatment may have good application prospects [16]. In particular, this system has many advantages. For example, the drug can be specifically transported to the specific location of a tumor and may achieve directional and focal inhibition of tumor cells as well as also causing less injury to normal cells, which may promote the highest treatment effects with minimal toxic effects on normal cells, resulting in increases in overall efficacy and safety as well as in patient compliance with chemotherapy [17]. The search for a safe and effective targeted drug carrier is the focus of ongoing research.

Drug carrier must meet the following conditions: firstly, the drug can be specific identified and transported through it. Secondly, it should have targeted performance, which means deliver drug to specific site combined with specific cell and ensuring the drug release. The targeting drug carrier studied widely nowadays include liposomes, nano materials, polymer materials, monoclonal antibodies, peptide etc [18]. Nano material carrier has certain adsorption and drug loading for some small molecule and peptide drugs with a quickly and sufficient targeted transport, but at the same time it can be adsorbed easily by normal tissue which resulted in certain poisonous effect [19]. Liposomes and polymer materials as targeted carriers may have targeted and specific defects [20]. Monoclonal antibody carrier accompany with a high cost and complicated process. What's more, the targeting effect of other carriers is not clear yet[21, 22, 23].

Professor AV Schally, a Nobel Prize winner at the Tulane University School of medicine, proposed the use of peptide as a carrier for targeted-therapy to delivery drug specific and relatively purposeful [24, 25]. A great deal of studies had found that some peptides existed in human's body, which highly expressed in tumor cells and tissues, while little in normal cells and tissues. The difference of peptide expression would be the promising approach for drug targeting therapy by peptide as carrier. More importantly, these peptides had no immunogenicity, which can be metabolized and cleared by human body with no harm [26].

In our previous study, we have proved that PEPT1 was highly expressed in HCC cells but relatively limited expressed in normal liver tissues. The specific expression of PEPT1 in HCC cells and tissues had been confirmed by Western blot, Real-time RT-PCR and immunohistochemistry in HCC. It is generally recognized that Ala-Lys-AMCA is a well-known PEPT1 substrate [27] and Caco-2 is an ideal model for studying the structure and function of PEPT1 [28]. We also verified that PEPT1 which expressed in HCC has the characteristic of oligopeptide transport activity through the results of D-Ala-Lys-AMCA uptake test in model cell Caco2 and HCC cells. Figure 1 showed some representative data and images, comprehensive results have been reported in another paper. Basing on the above findings, we speculate PEPT1 can be used as a carrier of HCC targeted chemotherapy. In this study we focus on exploring the feasibility of targeting PEPT1 to improve the antitumor efficacy of Doxorubicin in human HCC therapy.

The PEPT1 molecule recognizes a wide range of substrates with a certain structural features [29], such as di/tripeptide and their analogues [30]. In order to let Doxorubicin can be delivered by PEPT1, Doxorubicin-tripeptide conjugate was synthesized with Doxorubicin and a tripeptides − Gly-Gly-Gly which contained the structure of recognized and combined by PEPT1 [31]. The conjugate was successfully synthesized and identified with the purity of 98%.

We found that Doxorubicin-tripeptide conjugate can be transported into liver cancer cells as well as Caco-2 cells based on the emission of red fluorescence, and the longer the action time, the stronger the number and intensity of red fluorescence. Compared with Doxorubicin, Doxorubicin-tripeptide conjugate was more likely to enter the liver cancer cells with a strong fluorescence intensity under fluorescence microscopy and a high concentration by HPLC. These results suggested that the Doxorubicin-tripeptide conjugate can achieve a good uptake and absorption of the drug, of course through the PEPT1 carrier.

The effectiveness of drug targeted therapy, in addition to the specificity of drug carrier, is affected by environmental factors also. In order to determine the effect of concentration, pH, action time and inhibitor on the absorption of Doxorubicin, dynamic experiments, uptake experiment, substrate competitive inhibition experiment were proceeded.

The uptake of Ala-Lys-AMCA was time-dependent and concentration-dependent and affected by pH. Similar to Ala-Lys-AMCA, the uptake of Doxorubicin-tripeptide conjugate was also time-dependent and concentration-dependent, but not pH-dependent, the highest uptake of pH was 6.0. Its uptake was significantly reduced by inhibitors. Doxorubicin, however, did not have the above characteristics. On the other hand, these results proved that the expression of PEPT1 in HCC cells, and the Doxorubicin-tripeptide conjugate can indeed be transported into the liver cancer cells by PEPT1 to play a anti-tumor action simultaneously.

Small animal imaging techniques were adopted to evaluate the distribution of the drug in mice. We can see that Doxorubicin-tripeptide conjugate was mainly distributed in subcutaneous tumor tissue and less in heart tissue, which revealed specificity of this drug to tumor tissue. Part of the liver tissue also had certain fluorescence, these tissues was confirmed liver metastases by the pathology, which further verified the tumor target of Doxorubicin-tripeptide conjugate. In the control group of HepG2 mice, tumor tissue also showed high fluorescence intensity, maybe it was related to some experimental error, or maybe influenced by deficiencies of fluorescence imaging itself, for example weak sensitivity, the effect of background noise, the differences of exposure time and location, biological tissue light absorption and light scattering effect of their own and so on [32].

The antitumor effects of Doxorubicin-tripeptide conjugate were evaluated by MTT and nude mice tumor xenograft model experiments *in vitro* and *in vivo* respectively. Doxorubicin-tripeptide conjugate shows a better anti-cancer treatment with greater efficacy and lower toxicity, described as follows:

### Efficacy

In MTT test, Doxorubicin-tripeptide conjugate can inhibit the growth of two hepatocarcinoma cell lines and the inhibition rate achieved to 68.73%. The IC50 of Doxorubicin-tripeptide conjugate was higher than that of Doxorubicin. Since the Doxorubicin-tripeptide conjugate was synthesized by Doxorubicin and a tripeptide. The molecular weight of Doxorubicin-tripeptide is larger then that of Doxorubicin. The higer IC50 of Doxorubicin-tripeptide conjugate may correlate with the molecular weight difference between Doxorubicin-tripeptide conjugate and Doxorubicin. Furthermore, the PEPT1 transport activity also influence the delivery efficiency of Doxorubicin-tripeptide conjugate into HCC cells. *In vivo* test, according to the dose equivalent principle, we fed the tumor xenograft model mice with Doxorubicin-tripeptide conjugate 5 times dose than Doxorubicin. Doxorubicin-tripeptide conjugate can inhibit the increase of tumor volume and weight significantly, prolong the survival time and improve the quality of life of HCC mice. During the period of this drug, there was no decrease in food and water intake, some mice present weight and intake increased and with good spirit and activity.

### Toxicity

The main side effects of Doxorubicin were gastrointestinal symptoms, systemic symptoms, cardiac toxicity and bone marrow toxicity.

Firstly, with the development of HCC disease, mice appeared appetite and intake decreased, lethargy, weight loss gradually, reflecting the malignant consumption state of tumor. The above was more aggravated after Doxorubicin applied, but little existed in Doxorubicin-tripeptide conjugate group.

Secondly, compare the effects of drugs on the heart. The oxidation and antioxidation system of organisms are relatively balanced in physiological conditions. When oxygen free radicals produced too much and antioxidase activity decreased, destroy of this balance will lead to a series of lipid peroxidation reaction, eventually leading to cell damage and even death. This is also one of the important mechanisms of cardiac toxicity induced by Doxorubicin [33, 34]. GSH-PX was able to catalyze reduction reaction of reduced glutathione with hydrogen peroxide specifically, and remove harmful peroxide metabolites timely, thereby block a series of lipid peroxidation and protect structure and function of cell completely [35]. The activity of SOD indirectly reflects the ability of removing oxygen free radicals [36]. MDA is a degradation product of unsaturated fatty acid. Therefore, the level of GSH-PX, SOD and MDA is related to the severity of the cardiac disease, and also is a sensitive indicator of cardiac cell damage [37]. The more serious myocardial cell injury, the lower the serum levels of GSH-PX and SOD, and the higher myocardial enzyme and MDA level [38]. In this research, the amplitude of elevated myocardial enzymes, MDA and decreased GSH-PX, SOD were not obvious in the group of Doxorubicin-tripeptide conjugate, which suggested that this targeted-drug was less damage to the heart.

Finally, according to the peripheral blood and bone marrow smear test, Doxorubicin had obvious inhibitory effect on bone marrow, whereas this side effect can significantly reduced by the carrier of PEPT1, which transported Doxorubicin targeted to tumor tissue with little action to normal tissues.

Immunohistochemistry proceeded in tumor, liver, heart and jejunum tissues of mice revealed the positive expression of PEPT1 in tumor and liver metastasis tissues, little expression in healthy tissues, which was also provided the theoretical evidence for PEPT1 as the carrier of Doxorubicin. It was proved by *vivo* and vitro experiment that the safety and efficacy of Doxorubicin-tripeptide conjugate administrated for HCC through carrier PEPT1. Furthermore, PEPT1 can be used as a ideal carrier of doxorubicin in targeted therapy for HCC. In addition, all of these strategies in this manuscript should be verified by large sample research and further clinical applications.

In conclusion, this study will provide an experimental basis for further clarifying the function of the promising carrier PEPT1 and the efficacy of its targeted drug.

## MATERIALS AND METHODS

### Materials

The human hepatocarcinoma cell lines Bel-7402, HepG2 and the human colon cancer cell line Caco-2 were obtained from the Academia Sinica Cell Repository in Shanghai, China. Tissue chips were obtained from Xi’an Alina Biological Technology Co. Ltd, China. A rabbit polyclonal antibody to SLC15A1 (anti-PEPT1 antibody) was provided by Abcam. D-Ala-Lys-AMCA (2.5 mg/ml) was purchased from Biotrend Chemicals (Zurich, Germany), and Glycyl sarcosine (Gly-Sar), Glycyl glutamine (Gly-Gly) and Gly-Gly-Gly were obtained from Sigma-Aldrich. Healthy BALB/c-nu nude mice, female, (4.0 ± 0.2) weeks old, body weight (18.0 ± 2.0)g, purchased from the Institute of Laboratory Animal Science, Chinese Academy of Medical Sciences, animal Certificate No: SCXKA (Beijing) 2014-0004. Additionally, immunohistochemical reagents were supplied by Fuzhou Maixin Biotechnology Development Co. Ltd, China. Doxorubicin hydrochloride (HPLC purity 98.00%, Batch number130202) were gotten from Zhejiang Hisun Pharmaceutical Company Limited, China.

### Cell culture and animal feed

Bel-7402 cells were grown in RPMI-1640 (Gibco) medium containing 10% fetal bovine serum (FBS, Gibco), whereas HepG2 and Caco-2 cells were maintained in DMEM (Gibco) supplemented with 10% FBS, 1% 100 μg/ml penicillin (Sigma) and 100 μg/ml streptomycin (Sigma). All of the cells were incubated at 37°C in 5% CO_2_. Animals were raised in the animal center of Chinese Academy of Medical Sciences in SPF sterile laminar flow conditions with temperature (22–25°C) and humidity (45–50%). The research program was approved by the ethics committee of Tianjin Medical University General Hospital.

### Synthesis and identification method of Doxorubicin-tripeptide conjugate

Related reagents were obtained from Shanghai Top-Peptide Technology Co. Ltd, China. The synthesis procedures were as follows:

(1) Placed 0.8 g 2-cl-trt Resin (degree of substitution 1.0 mmol/g) into a clean and dry reaction tube, added Deoxycytosine methylase (DCM) swelling for 15 min and then filtered DCM. Added Fmoc-glycine-OH (Fmoc-Gly-OH) into the reaction tube, which was 1.5 times as much as the resin. Added DCM again for dissolution and then added N,N-Diisopropylethylamine (DIEA) (10 times the equivalent) for nitrogen bubbling reaction 2 h, and then added methanol reacted for 30 min directly. In the end washed 6 times with appropriate amount of Dimethyl Fumarate (DMF). 20% piperidine/DMF solution was added twice and reacted for 10 min, 5 min respectively to take off the Fmoc of amino acid. After that it was washed with DMF 4 times and with methanol 2 times.
(2) Detected the product of step (1) by ninhydrin method then continue to next step.
(3) Weigh 3 times equivalent Fmoc-Gly-OH (the second amino acid at C-terminal) and ONNN’N’ tetramethyluronium hexafluophosphate (HBTU), put them into reaction tube, added DIEA 0.5 ml, reacted for 40 min, washed by DFM 4-6 times.
(4) Detected resin samples by ninhydrin method, After the reaction showed white colour then added piperidine to wash Fmoc 10 min, 5 min respectively. Washed in turn, DMF 4 times, methanol 2 times.
(5) Detected the product of step (4) by ninhydrin method then continue to next step.
(6) Weigh 3 times equivalent Fmoc-Gly-OH (the third amino acid C-terminal) and repeated step (3) and then detected the product by ninhydrin method then continue to next step.
(7) Put the resin in the reaction tube into 50 ml centrifuge tube, Added 4% glacial acetic acid /DCM solution 15 ml, shake for 2 h, take the filtrate, spin and dry. Added 5 times equivalent HBTU and 10 times equivalent DIEA, with DCM as solvent, reacted for 40min to activate the carboxyl of poly peptide chain. Then added 1.2 times equivalent Doxorubicin and 2 times equivalent DIEA reacted for 3 h avoiding light.
(8) Spin dry the reaction solution, added 40% trifluoroacetic acid (TFA), reacted for 1.5 h to remove the protection group of T-butyloxycarbonyl (BOC).
(9) After precipitation by ether and centrifugation, the crude product produced.
(10) The crude product was then purified by HPLC and MS. The qualified targeted product (Doxorubicin-tripeptide conjugate) was frozen and dried by freeze drying machine, then packed avoiding light.

### Proliferation inhibition assay by MTT

Cells in the growth log phase were seeded in 96-well plate (200 ul/well) with density 8×10^3^/well for 24 hours, then the drugs were added. The concentration of Doxorubicin-tripeptide conjugate was 0.005 mg/ml, 0.01 mg/ml, 0.02 mg/ml, 0.04 mg/ml, 0.06 mg/ml in turn, while Doxorubicin followed by 0.005 mg/ml, 0.01 mg/ml, 0.02 mg/ml, 0.04 mg/ml, 0.06 mg/ml with a same dosing volume 100 μl. Each concentration group included five equal wells and was provided with a control well of medium alone as a negative control. Add 20 μl of MTT Reagent ( 5 mg/ml, Sigma) to each well, including controls and continue to be incubated at 37°C for 4 hours. Remove the supernatant, drop plus DMSO (200 μl/well) and measure the OD value in each well, including the blanks, at 490 nm by a Multi-Mode Reader (BioTek, Synergy 2, USA). To calculate the inhibitory rate and the half maximal inhibitory concentration (IC50) according to the formula: Inhibitory rate =(1-( the dosing group OD/the control group OD )) × 100%, IC50 = lg^-1^ (Xm-i (ΣP-0.5)) (Xm: the logarithm of maximum concentration, I: the logarithm of maximum dose/ adjacent dose, P: the sum of each group inhibitory rate, Pm: the maximum inhibitory rate, Pn: the minimum inhibitory rate ).

### Fluorescence microscope Observation

All cell lines were cultured in 6-well plates for one day, after the cells were adherent to the plate aspirated the culture medium and washed with PBS twice. Doxorubicin-tripeptide conjugate (0.05 mg/ml) and Doxorubicin (0.01 mg/ml) were added and incubated avoid light at 37°C for 15 min, 30 min, 1 h, 2 h, 3 h respectively. Then the cells were washed again with PBS and observed finally under the Fluorescence microscope (OLYMPUS IX51).

### HPLC analysis

When the density of HCC cells reached to 3 × 10^6^/ml, they were trypsinized and centrifuge (4°C, 800rpm, 5min), then discarded the supernatant, dropping medium 3 ml and evenly mixed. Cell suspension was divided into three groups (control group, Doxorubicin-tripeptide conjugate group and Doxorubicin group). The drug concentration was 0.01 mg/ml for cultured 2 hours avoiding light. Then the cells obtained by centrifugation were proceeded by the following steps: washed 3 times with 1 ml normal saline, added 0.5 ml ultra pure water (Millipore), broken by ultrasonic wave, drooped in acetonitrile (Tianjin Concord Technology Co. Ltd, China) 0.5 ml, mixed, ultrasonic oscillation for 10 mins, centrifuge (1200 rpm, 15 min), take the supernatant, filtration with filter membrane, take 20 μl for HPLC analysis (Agilent 1100 HPLC system, USA).

Chromatographic conditions were as follows: chromatographic column: DIONEX Acclaim 120 C18 (2.1 × 250 mm, 5 μm 120A), Mobile phase: Acetonitrile-0.2%NaH_2_PO_4_ aqueous solution (35: 65), Velocity: 0.35 ml/min, column temperature: 30°C, DAD, 254 nm, sample size: 20 μl. 1 mg control article of Doxorubicin-tripeptide conjugate and Doxorubicin were mixed with 1 ml acetonitrile (50%) respectively, then were diluted by stock solution (1 mg/ml ) to 10 μg/ml, 5 μg/ml, 2 μg/ml, 1 μg/ml, 0.5 μg/ml, 0.2 μg/ml, 0.1 μg/ml, 0.05 μg/ml. To analyze by HPLC and draw standard curve according peak area. According to the chromatographic condition, the retention time (tR), peak position time, chromatographic behavior, resolution (R), the minimum detectable concentration and the intracellular drug concentration of these two drugs were detected in turn.

### Uptake Studies

The transport of Doxorubicin-tripeptide conjugate by PEPT1 was evaluated by time course, dynamic and competitive inhibition tests. The uptake study was performed on the confluent cell monolayer as described previously [8, 9].

The uptake buffer was HBSS buffer (0.952 mM CaCl_2_, 5.36 mM KCl, 0.441 mM KH_2_PO_4_, 0.812 mM MgSO_4_, 136.7 mM NaCl, 0.385 mM Na_2_HPO_4_, 25 mM D-glucose, and 10 mM HEPES) adjusted to pH 6.0. First, the time course test was observed at the same pH value (6.0) with 0.05 mg/ml Doxorubicin-tripeptide conjugate at different time points (0, 15, 30, 60, 120 and 180 min). Then four different initial concentrations (0.025 mg/ml, 0.05 mg/ml, 0.10 mg/ml, 0.15 mg/ml) of Doxorubicin-tripeptide conjugate were incubated with HCC cell monolayer at pH 6.0 for 120 min. At last, 0.05 mg/ml Doxorubicin-tripeptide conjugate was fed to HCC cells at different pH uptake buffer (5.4, 6, 7.4 and 8.4) for 120 min. Inhibition tests were conducted by preincubating the cells with competitive compounds Gly-Sar, Gly-Gln, Gly-Gly-Gly (0.01 mg/ml) respectively for 30 mins and then incubated with Doxorubicin-tripeptide conjugate (0.05 mg/ml) for 1 h. After removing the buffer and rapidly washing 3 times with ice-cold PBS, the cellular uptake was specifically examined with a Multi-Mode Reader (BioTek, Synergy 2, USA) with Excitation 485/20 and Emission 590/35.

### Animal studies

### Nude mouse tumor xenograft model

An animal model of HCC cells (Bel-7402, HepG2) transplanted into nude mice was established according to the method described in ref. 10.

### Group-division and treatment

After the HCC mice model was established, the nude mice were randomly assigned to three groups: Group A (control group: administration with normal saline 0.2 ml every time), group B (administration with Doxorubicin-tripeptide conjugate) and group C (administration with Doxorubicin). Meanwhile administration was divided into low dose group (HepG2) with Doxorubicin 40 mg/m^2^ and Doxorubicin-tripeptide conjugate 200 mg/m^2^ and high dose group (Bel-7402) with Doxorubicin 60 mg/m^2^ and Doxorubicin-tripeptide conjugate 300 mg/m^2^ by rat tail vein [10]. Treatment was conducted every 3 weeks for 5 weeks. Pay close attention to the general condition of the mice and their subcutaneous tumor and draw sur*vivo*rship curve. The mice were weighed every 3 days and tumor volume was measured with a digital caliper using the formula: volume = (1/6).π. length × width × height. At the end of the experiment, tumor weight and volume was measured to obtained the tumor inhibitory rate and actual volume, which were calculated by the follow formula respectively: tumor inhibitory rate = (The average tumor weight of control group-experimental group)/control group × 100 %. volume = (1/6).π. length × width × height.

### Biochemistry analysis

After the course of administration, mice were sacrificed, then the blood was collected to detect relevant indexes including quantitative serum protein, blood routine, myocardial enzymes (CK, CK-MB, LDH, AST), GSH-PX, MDA and SOD, the bone marrow taken to make bone marrow smear. What's more, the tumor, liver, jejunum and heart tissue of mice were dissected firstly for Small Animal Imaging (KODAK IS in-*vivo* FX, Kodak), and secondly were placed in the 10% Formaldehyde solution to prepare paraffin section, HE staining for histological examination. Lastly, the expression of PEPT1 in each tissue was detected by immunohistochemical SP method. The tests of CK, CK-MB, LDH, AST, GSH-PX, MDA and SOD were completed in accordance with instructions strictly (Jiancheng Bioengineering Institute, Nanjing, China).

### Statistical analysis

Significance of Kaplan-Meier statistics was tested by calculating the log-rank. Data were expressed as the mean ± standard deviation (mean ± SD). SPSS version 16.0 (SPSS, Chicago, IL, USA) was used for all calculations. Statistical significance was determined at *P* < 0.05.

### Ethical standards

All procedures performed in studies involving human participants were in accordance with the ethical standards of the research committee of Tianjin Medical University General Hospital, China. All participants gave written informed consent before participating in the study. The research program was approved by the ethics committee of Tianjin Medical University General Hospital, China.
